# User Perceptions of Different Vital Signs Monitor Modalities During High-Fidelity Simulation: Semiquantitative Analysis

**DOI:** 10.2196/34677

**Published:** 2022-03-18

**Authors:** Samira Akbas, Sadiq Said, Tadzio Raoul Roche, Christoph B Nöthiger, Donat R Spahn, David W Tscholl, Lisa Bergauer

**Affiliations:** 1 Institute of Anesthesiology University of Zurich University Hospital Zurich Zurich Switzerland

**Keywords:** avatar, patient monitoring, semiquantitative research, simulation study, situation awareness, user-centered design, visual-patient-avatar

## Abstract

**Background:**

Patient safety during anesthesia is crucially dependent on the monitoring of vital signs. However, the values obtained must also be perceived and correctly classified by the attending care providers. To facilitate these processes, we developed Visual-Patient-avatar, an animated virtual model of the monitored patient, which innovatively presents numerical and waveform data following user-centered design principles. After a high-fidelity simulation study, we analyzed the participants’ perceptions of 3 different monitor modalities, including this newly introduced technique.

**Objective:**

The aim of this study was to collect and evaluate participants’ opinions and experiences regarding 3 different monitor modalities, which are Visual-Patient-avatar, Split Screen (avatar and Conventional monitor alongside each other), and Conventional monitor after using them during simulated critical anesthetic events.

**Methods:**

This study was a researcher-initiated, single-center, semiquantitative study. We asked 92 care providers right after finishing 3 simulated emergency scenarios about their positive and negative opinions concerning the different monitor modalities. We processed the field notes obtained and derived the main categories and corresponding subthemes following qualitative research methods.

**Results:**

We gained a total of 307 statements. Through a context-based analysis, we identified the 3 main categories of “Visual-Patient-avatar,” “Split Screen,” and “Conventional monitor” and divided them into 11 positive and negative subthemes. We achieved substantial interrater reliability in assigning the statements to 1 of the topics. Most of the statements concerned the design and usability features of the avatar or the Split Screen mode.

**Conclusions:**

This study semiquantitatively reviewed the clinical applicability of the Visual-Patient-avatar technique in a high-fidelity simulation study and revealed the strengths and limitations of the avatar only and Split Screen modality. In addition to valuable suggestions for improving the design, the requirement for training prior to clinical implementation was emphasized. The responses to the Split Screen suggest that this symbiotic modality generates better situation awareness in combination with numerical data and accurate curves. As a subsequent development step, a real-life introduction study is planned, where we will test the avatar in Split Screen mode under actual clinical conditions.

## Introduction

Although perioperative mortality directly attributable to anesthesia is low in high-income countries and has significantly declined over the last 50 years, the World Health Organization describes anesthesiologic and surgical complications as the leading cause of preventable perioperative morbidity and mortality [1-4].

Among all anesthesia complications leading to permanent brain damage or death, two-thirds are caused by inaccurate situation awareness. This concept developed by Mica Endsley comprises a chain of information processing including the three core levels of perception (level 1), comprehension (level 2), and projection (level 3), whereby level 1 is most frequently affected [5-7]. For appropriate decision-making and thus avoiding errors, a situation must be recognized, its severity assessed, and the correct next steps taken while anticipating future progress. This cognitive process is influenced by individual factors such as experience and environmental resources. Well-established methods such as perioperative checklists were developed with the intention of improving environmental resources [8]. In addition, new tools are needed to impact situation awareness positively and thus reduce perioperative anesthesiologic complications.

Hence, we developed Visual-Patient-avatar as a beneficial environmental factor on situation awareness in patient monitoring. This avatar-based visualization on a patient monitor displays an animated model of the measured numerical parameters combining principles of logic and user-centered design [9]. Previous computer-based studies have shown that more vital signs were observed when using this new technique, subjective diagnostic confidence increased, and perceived workload declined compared to conventional patient monitoring [10-12]. However, the use of Visual-Patient-avatar in a high-fidelity simulation study has not yet been analyzed, including its qualitative aspects.

This study aims to collect and assess the opinions and experiences of participants concerning the three different patient monitoring modalities, which are (1) Visual-Patient-avatar, (2) Split Screen (avatar and conventional patient monitoring side by side), and (3) conventional, after using them in simulated critical anesthesia events [13]. We sought to capture the advantages and disadvantages of the different monitor settings to foster the avatar’s development and, in the future, facilitate its implementation in everyday clinical practice.

## Methods

### Approval and Consent

The Cantonal Ethics Committee of Zurich in Switzerland issued a declaration of no objection after reviewing the study protocol (Business Management System for Ethics Committees Req-2020-00059). All participants signed written informed consent for the use of their data for research purposes and participated voluntarily without any financial compensation.

### Study Design

This is a researcher-initiated, single-center, semiquantitative study investigating physicians’ and nurses’ perceptions of using Visual-Patient-avatar in simulated critical anesthesia events. We conducted this study at the University Hospital of Zurich in Switzerland, in May 2020. We included the same 104 care providers grouped in 52 teams of a recently published study that evaluated avatar-based patient monitoring in a high-fidelity simulation study [13].

### Previous Avatar-Based Patient Monitoring Simulation Study and Participant Interviews

**Figure 1:**
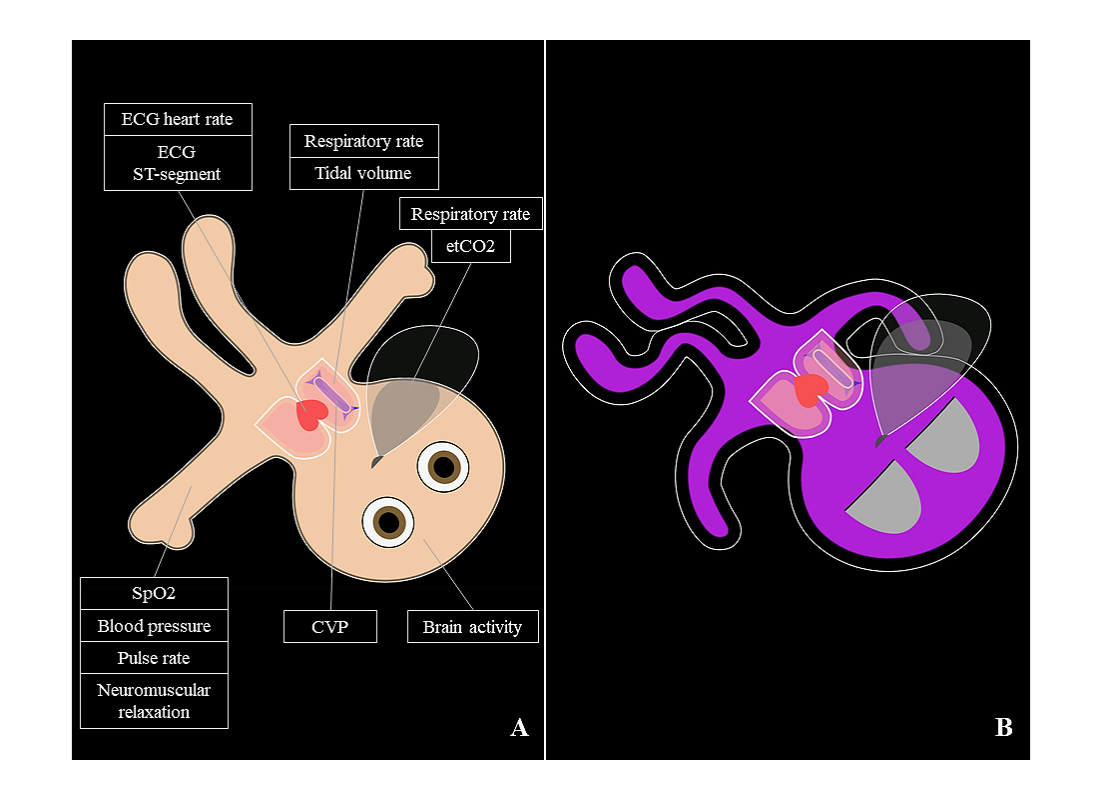
Two examples of Visual-Patient-avatar used during the high-fidelity simulation. CVP: central venous pressure; ECG: electrocardiogram; etCO2: end-tidal carbon dioxide; SpO2: peripheral oxygen saturation; ST: ST-segment.

This recently published study showed noninferiority of Split Screen compared with Conventional monitoring for performance during anesthesia crisis events. The probability of communicating the correct reason for the emergency was increased using the Visual-Patient-avatar as the monitor modality [13]. Figure 1 shows the 13 available vital signs and an example with possible deviations and additionally. Part (A) depicts an awake patient with vital signs within normal range. The avatar’s body pulsates during patient monitoring, whereby the frequency and extension indicate the pulse rate and blood pressure, respectively. In part (B), we demonstrate a desaturated (purple color), deeply sedated (eyes closed) patient with muscle relaxation (floppy extremities). Hypotension is represented by the gap between the purple body and the white boundary line. If the body temperature leaves the normal range, ice crystals or heat waves become visible around the avatar. Additionally, Multimedia Appendix 1 provides an animated version of the 2 examples.

**Figure 2:**
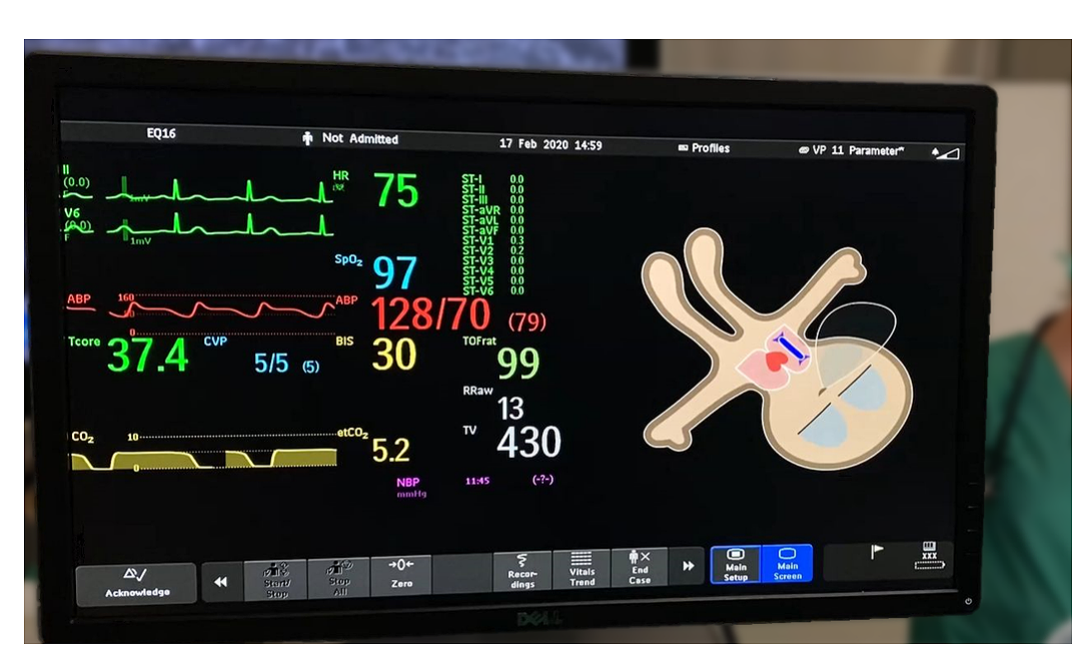
Example of a Split Screen display during simulation with the Conventional monitor on the left and the Visual-Patient-avatar on the right side. The beige skin tone corresponds to a normal peripheral oxygen saturation, and closed eyes imply a sedated patient.

After a short briefing and a training scenario, the participants completed 3 different emergency scenarios, each with 1 of the following 3 different monitor modalities: only Visual-Patient-avatar, Split Screen, and only conventional, number-based and waveform-based monitor. Figure 2 illustrates an example of a Split Screen display during simulation, and the video in Multimedia Appendix 2 shows a recording of a simulation scenario. After completing all scenarios, we asked the following 2 questions: “What do you like about the monitor settings? Eg, particular strengths?” and “What do you dislike about the monitor settings? Eg, potential problems, limitations?” The study authors TRR and SS recorded the participants’ responses as field notes on an iPad (Apple Inc). The participants reviewed the final field note transcripts, modifying or adding to them if warranted.

### Semiquantitative Analysis

**Figure 3:**
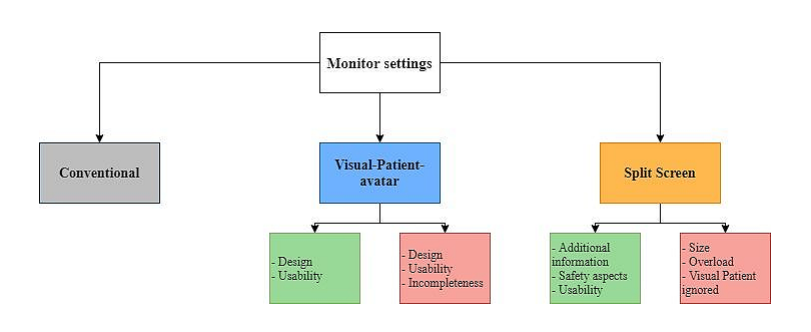
Hierarchical coding tree concerning user perceptions of the 3 different monitor modalities. The green boxes include positive subthemes of the respective major topic. The red boxes contain negative subthemes of the corresponding major topic.

After collecting all answers, we translated them from German to English using an online translating service, Deepl (DeepL GmbH). In Multimedia Appendix 3, we provide the complete translated field notes. There were no comments made from 12 participants. To gain a first impression by identifying frequently mentioned terms, we excluded filler words such as “and” or “the” and performed a word count using Microsoft Word (Microsoft Corporation). Although the word count does not provide information about the content of individual statements, this approach helped us to identify similar expressions. Subsequently, we grouped the statements using the template approach, identified main topics, and generated a coding tree, which we modified until all essential and frequent statements could be classified [14,15]. According to the recommendations of reporting qualitative research, study authors SA and LB, who were not involved in the interview process, evaluated the statements independently of each other, using the final coding tree displayed in Figure 3, which was created using draw.io (Seibert Media GmbH) [16-18]. Before determining a joint code in case of disagreement, we calculated the interrater reliability to validate the rating.

### Statistical Analysis

We report the number of statements and their percentages relating to the superior topics. To manage our data and to generate the figures, we used Microsoft Word and Microsoft Excel. To quantify the interrater reliability, when assigning the individual statements to a particular topic of the final coding tree, we calculated the Cohen kappa using R, version 4.0.5 (R Foundation for Statistical Computing) [19]. To estimate percent agreement, we used Microsoft Excel.

## Results

### Participant and Field Notes Characteristics

We acquired field notes between May 4, 2020, and May 28, 2020. All participants were employees of the University Hospital of Zurich. Of the total 104 participants, 92 (88%) senior physicians, resident physicians, nurse anesthetists, and trainee nurse anesthetists took part in the interview process right after completing the simulation scenarios. Table 1 provides a detailed description of the study and participant characteristics.

Analyzing the field notes obtained, we identified 329 individual statements consisting of 2807 words. Of 329 statements, 22 (7%) were not comprehensible to us in terms of content even after several discussions, so we classified them as “not codable.” Statements in this category included subjective opinions such as “I like it” (participant #13.1). The remaining 307 statements were analyzed semiquantitatively, which allows the calculation of the proportions of individual statements among the main topics and subthemes without applying statistical tests [20,21]. Overall, the ratio of statements to the question, “What do you like about the monitor settings” (144/307, 47%) compared to the negative perceptions (163/307, 53%) was balanced.

**Table 1:** Study and participant characteristics in detail (n=92).

Study and participant characteristics	Values
Participants who submitted field notes, n (%)	92 (88)
Female participants, n (%)	46 (50)
Senior physicians, n (%)	14 (15)
Resident physicians, n (%)	33 (36)
Nurse anesthetists, n (%)	30 (33)
Trainee nurse anesthetists, n (%)	17 (16)
Total anesthesia experience (years), mean (IQR)	6.6 (1.5-8)

### Semiquantitative Analysis

Beginning our semiquantitative analysis, we performed word counts to expose potential main themes. The analysis revealed that “Visual Patient” was the most frequently occurring term in the field notes obtained to answer both the positive (35 times in 144 statements) and negative questions (38 times in 163 statements).

**Figure 4:**
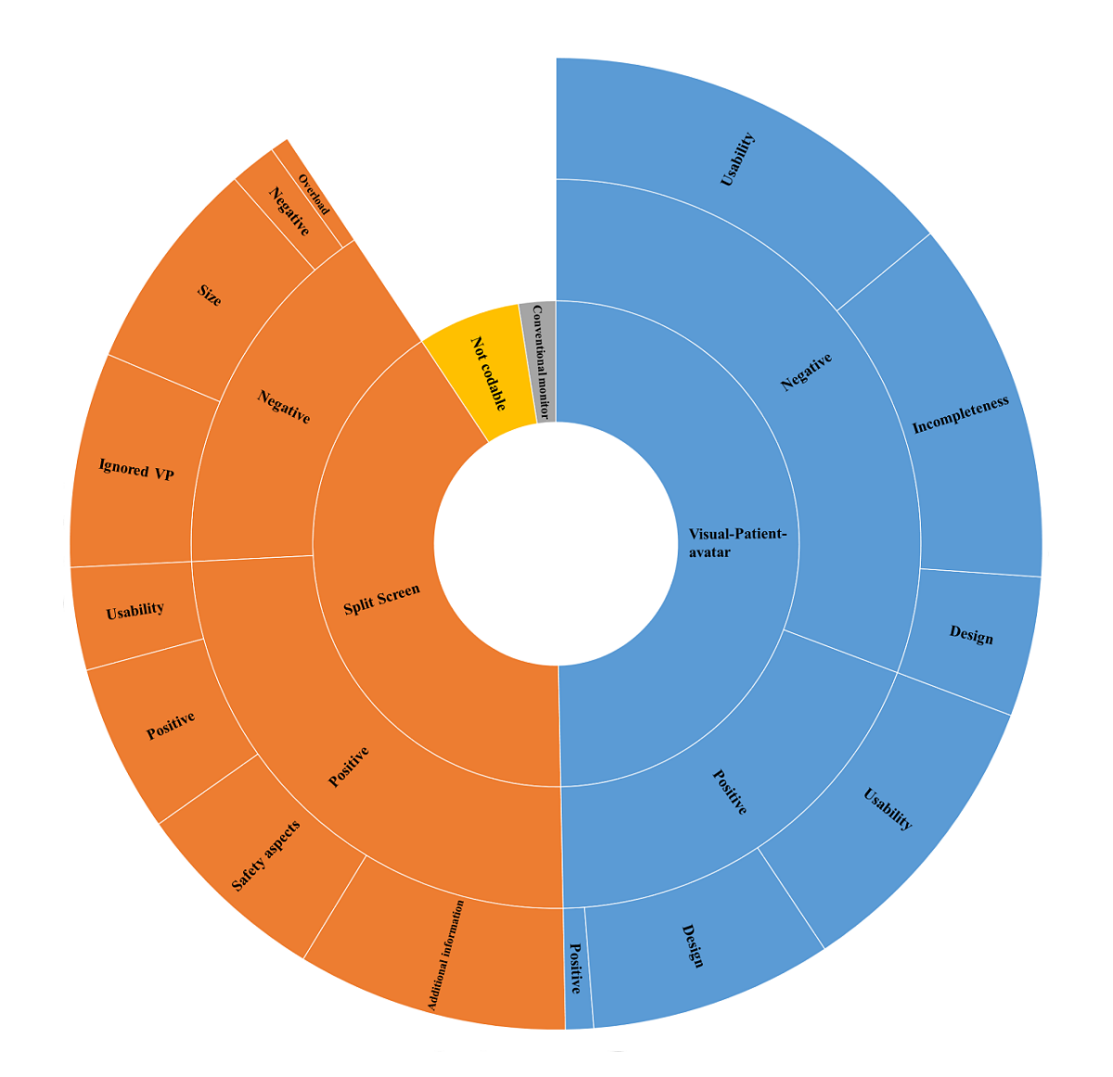
Sunburst diagram to reflect the user perceptions of the 3 different monitor settings. The width of a section represents the respective percentage of the topic on all given statements (N=307). Ignored VP: ignored Visual-Patient-avatar.

Based on using qualitative research methods and testing 3 monitor modalities, the final coding tree contains the 3 main categories of Visual-Patient-avatar, Split Screen, and Conventional monitor, as well as 4 main topics with 11 subthemes. When independently assigning all 327 statements received to 1 of these topics, the study authors SA and LB achieved 80% interrater agreement with a substantial Cohen kappa of 0.78 [22]. In the case of differently coded statements, a review and joint assignment followed to achieve 100% interrater agreement after the second round of coding. Figure 4 visualizes the percentage distribution of all statements among the different categories. The 3 main categories are located in the innermost circle. The associated major topics and subthemes are displayed hierarchically toward the outside. Table 2 outlines the major topics with examples. In the subsequent sections, we describe the individual categories in detail with percentages and examples. The calculations refer to the codable statements.

**Table 2:** The major topics with participant count, percentages, and examples.

Major topics and subthemes			Examples
**Visual-Patient-avatar positive (61/307, 20%)**			
	Design	Especially oxygenation and body temperature well displayed. [#^a^11.2] Integration of all values on one avatar. [#47.2]	
	Usability	Information simplified by Visual-Patient-avatar. [#13.2] Overview of relevant parameters at a glance through Visual-Patient-avatar. [#25.2]	
**Visual-Patient-avatar negative (99/307, 32%)**			
	Design	The blood pressure feature was not easy to understand for me. [#24.2] Visual-Patient-avatar: head too large in contrast to heart and lung. [#25.1]	
	Usability	Visual-Patient-avatar takes some time getting used to; not entirely intuitive at first. [#29.2] At the moment still difficult but with potential. [#38.1]	
	Incompleteness	Numbers and ranks are missing. [#10.1] Lacking information quantification with the Visual-Patient-avatar. [#48.1]	
**Split Screen positive (79/307, 26%)**			
	Additional information	Additional information by Visual-Patient-avatar. [#14.2] I like the combination of new and old monitoring. [#18.2]	
	Safety aspects	With Visual-Patient-avatar changes faster visible than with numbers or curves. [#13.1] More safety. [#41.1]	
	Usability	Split monitoring helps to focus. [#10.1] I prefer the split monitor, Visual-Patient-avatar as first initial diagnosis—quantification via Conventional monitoring. [#16.1]	
**Split Screen negative (60/307, 20%)**			
	Size	Info partly displayed a bit small. [#13.2] Needs appropriate monitor size. [#24.1]	
	Overload	Too much information at once in the emergency situation. [#12.2] Screen very full. [#30.2]	
	Visual-Patient-avatar ignored	Looked at numbers. [#9.1] I barely looked at the Visual-Patient-avatar. [#18.2]	
Conventional monitor (8/307, 3%)			I want to see the details or parameter more precisely. I prefer the “usual” monitor view. [#26.1]

^a^Participant number.

### Statements About Visual-Patient-Avatar

We assigned 160 of 307 (52%) statements to the main category Visual-Patient-avatar. Through inductive free coding, the 2 major topics, Visual-Patient-avatar positive (61/307, 20%) and Visual-Patient-avatar negative (99/307, 32%), were revealed.

We divided the positive major topic into the 2 subthemes, design (26/307, 8%) and usability (32/307, 10%). Concerning design features, the participants distinguished the simplified (participant #13.2) and realistic (participant #17.1) appearance of the avatar. Participant #11.2 outlined that “Especially oxygenation and body temperature is illustrated well.” The participants also recognized advantages in terms of usability. They found that Visual-Patient-avatar is “Intuitively understandable” (participant #31.1), “Gives a good overview” (participant #34.1), and helps to grasp the situation quickly (participant #37.1). We allocated more common statements such as “Integration of all values on one avatar” (participant #47.2) to the major topic Visual-Patient-avatar positive (3/307, 1%).

Regarding negative properties of the avatar, the participants’ responses depicted design (15/307, 5%), usability (45/307, 15%), and incompleteness (39/307, 13%) as subthemes. For participant #43.2, the thorax displayed too small, and the vena cava representation was unclear. Others raised concerns about possible misinterpretations (participant #46.1) because of the unfamiliar (participant #48.2) and confusing (participant #48.1) vital sign presentation within Visual-Patient-avatar technique. Without concrete values (participant #35.2) and curves such as the electrocardiogram (participant #42.2), the avatar did not help in solving the emergency scenarios.

### Statements About the Split Screen

In 139 of 307 (46%) statements, the participants noticed this main category, which we classified into the major topics Split Screen positive (79/307, 26%) and Split Screen negative (60/307, 20%).

In interrater consent, the positive major topic included the 3 subthemes additional information (29/307, 9%), safety aspects (21/307, 7%), and usability (11/307, 4%). Through “Increasing attention” (participant #10.1), “Faster recognition of changes” (participant #29.2), and the “Quick overview” (participant #45.2), the participants perceived a higher level of safety. Several participants found the Split Screen mode overall “Helpful” (participant #47.1) and “Effective” (participant #17.1) in its use. We allocated responses that generally considered the combination advantageous to the major topic, positive Split Screen (18/307, 6%).

The negative major topic concerning Split Screen enclosed the 3 subthemes, “size,” “overload,” and “Visual-Patient-avatar ignored.” These were named, respectively, in 23/307 (7%), 23/307 (7%), and 9/307 (3%) statements. The participants criticized the small display and thus the difficulty of detecting details of the curves (participant #20.1) and the Visual-Patient-avatar (participant #21.2). Furthermore, they claimed the Split Screen to be crowded (participant #30.2), and that there is “Too much information at once in the emergency” (participant #12.2). In addition, the analysis of the field notes discovered that several participants ignored the avatar. General annotations such as “Not sure about the added benefit” (participant #48.1) were assigned to the major topic, negative Split Screen (5/307, 2%).

### Statements About the Conventional Monitor

A small number of the field notes referred to the main category Conventional monitor (8/307, 3%). Some participants just stated that they “Prefer the usual monitor view” (participant #26.1). Furthermore, we grouped responses that mentioned the familiar audio support in this main category.

## Discussion

### Principal Findings

This semiquantitative single-center study explored the impressions of anesthesia personnel when using the existing Conventional monitor compared with the new modality Visual-Patient-avatar—either the avatar only or the Split Screen variant. User perceptions can uncover improvement opportunities, and their consideration is essential for the success of new medical techniques. We assigned most of the statements to the main category Visual-Patient-avatar, highlighting positive characteristics and negative features such as the absence of quantitative data. Many annotations also evaluated the Split Screen modality, while only a few participants commented on the well-known Conventional monitor. The latter seems coherent as Visual-Patient-avatar is a novelty and thus attention catching*.*

The avatar’s development was guided by the idea of providing a monitor tool that improves situation awareness through its user-centered design principles. Following the definition of a user-centered design through Mica Endsley [9], many participants stated the avatar technique to include beneficial design and usability features such as being simplified and intuitive (positive Visual-Patient-avatar: 61/307, 20%). When used with the Conventional monitor in Split Screen mode, Visual-Patient-avatar increases attention and provides a quick overview. Possible changes in vital signs can then be quantified using the conventional display (safety aspects: 21/307, 7%). The aspect of time saving through faster detection is essential in patient care, as for example, postoperative renal dysfunction is related to the overall duration of hypotension during general anesthesia [23].

Many participants claimed the missing numbers and curves when using only the avatar makes a more precise diagnosis impossible (incompleteness: 39/307, 13%). In Visual-Patient-avatar, the data for each vital sign is preprocessed to show different states (no data, too low, normal, or too high), aiming to reduce complexity. We understand the technology as a supplement, which cannot replace the Conventional monitor; however, it can improve care providers’ situation awareness by presenting information that is easy to perceive and comprehend.

On the question, “What did you dislike about the monitor settings?”, this analysis found that size (23/307, 7%) and overload (23/307, 7%) were the main critical points concerning the Split Screen mode. During the simulation study, the scenarios run on 12-inch patient monitors (Philips IntelliVue MX500; Koninklijke Philips NV, Amsterdam, The Netherlands). However, the technique for the real-life clinical implementation is compatible with the Philips IntelliVue MX 550 monitor, which offers a larger display of 15 inches. This fact can mitigate the criticism, but it is known that a high information load can have a detrimental effect on the ability to set priorities and can confuse the individual [24]. This would contradict the basic idea of Visual-Patient-avatar and must be kept in mind.

The impression of an overload could also occur because the technique of the Visual-Patient-avatar and its implementation as Split Screen variant is new and therefore cognitively demanding. Accordingly, several participants mentioned being unfamiliar with the avatar, whether used individually or in Split Screen mode. Upon introduction of the new technique into clinical routine, all users will receive education and training lessons. Nevertheless, it will take time to get used to the new monitor modalities and fully implement them mentally, as especially very experienced care providers have been working with the Conventional monitor modality for decades. The successful implementation of new techniques can be demonstrated by the sonographically guided insertion of central venous catheters [25]. After initial skepticism, this method is nowadays preferred both in the literature and clinically, as the complication rate is lower than with landmark-guided puncture [26].

To achieve a high level of user acceptance, an intuitive interface and cognitive ease are crucial points [27]. Visual-Patient-avatar presents the information close to clinical reality. For example, the avatar’s skin turns purple in case of hypoxemia, or its eyes are open when the brain-activity sensor detects a high signal. The participants’ appreciation of the realistic and clear vital sign display (design: 26/307, 8%) is in line with the results of the study by Wachter and colleagues [28], which shows that an anatomically related interface is particularly intuitive. However, together with design and technical specialists from Philips (Koninklijke Philips NV), an intensive redesign process was carried out to improve weaknesses in the design, such as the vena cava display. Even though some steps are still needed until clinical introduction, we expect visualization techniques to have a great future in medicine after this study. It is encouraging that Hamilton Medical AG provides the “dynamic lung” in ventilators to visualize specific lung parameters [29].

### Strengths and Limitations

This study has several limitations. In qualitative and semiquantitative analysis, the structure and results are developed inductively and cannot be applied to a broader population as it does not investigate statistical significance. Nevertheless, this approach allowed us to gain firsthand perceptions and experiences from our participants right after using the different monitor modalities. Generally, a nonquantitative assessment stays close to the participants’ point of view, implying a certain subjectivity [30,31]. However, this is put into perspective by the high number of participants and their diversity. As a single-center study, possible selection bias cannot be excluded. It is conceivable that the results vary under different circumstances.

To date, only computer-based studies have been conducted with the Visual-Patient-avatar [10,11]. One of the strengths is the high-fidelity simulation, which made it possible to test the new technique realistically during anesthesiologic emergency scenarios and to derive conclusions for its use under clinical conditions [32]. Thus, we obtained the first opinions on Visual-Patient-avatar directly after experiencing the urgency of emergent patient treatment. These findings greatly impact the further development of the technique up to the point of clinical implementation.

### Conclusion

We designed this study to determine care providers’ perceptions concerning monitor modalities incorporating the Visual-Patient-avatar technique. One of the key findings was that the participants experienced the avatar technique’s underlying design principles and characteristics positively under active use in the context of a high-fidelity simulation. This insight complements those of earlier studies using the Visual-Patient-avatar technique in computer-based studies [33]. The participants confirmed the value of the Split Screen mode through its combination of visual impressions and simultaneous quantification with numerical parameters. This monitor variant, planned for future clinical implementation, gives a quick overview and draws attention to changes specified by the conventional part. The next step in the development is a planned real-life introduction study of the avatar in Split Screen mode under actual clinical conditions. This modality’s weakness, based on the large amount of information displayed, will be reflected in the further planning process and will be reviewed through future studies. By testing the avatar in a simulated clinical environment for the first time, we are taking a significant step toward our vision: to help care providers in situations of high cognitive load to better prioritize information and thus positively influence decision-making for the patient’s benefit.

## Appendix

Multimedia Appendix 1 Video with two examples of an animated Visual-Patient-avatar.

Multimedia Appendix 2 Video of simulations scenario presenting Visual-Patient-avatar and Conventional monitor.

Multimedia Appendix 3 The translated field notes of 92 participants. The brackets [] indicate which parts were each assessed as one statement.
