# Supplementary material for: User Perceptions of Different Vital Signs Monitor Modalities During High-Fidelity Simulation: Semiquantitative Analysis
Source: JMIR Hum Factors. 2022 Mar 18;9(1):e34677. doi: 10.2196/34677 (PMC8976258; doi:10.2196/34677)
Supplement: Multimedia Appendix 3 [file humanfactors_v9i1e34677_app3.docx]

**Translated participants’ field notes**

Translated field notes of 92 participants of this study. From 12 participants exist no comments. The brackets [] indicate which parts were each assessed as one statement.

**Participant #7.1**

POSITIVE: What did you LIKE about the monitor settings? E.g., particular strengths?

[CO2 very clear ][and distinct]

NEGATIVE: What did you DISLIKE about the monitor settings? E.g., potential problems, limitations?

[Nothing]

**Participant #7.2**

POSITIVE: What did you LIKE about the monitor settings? E.g., particular strengths?

[Pretty good] [Faster]

NEGATIVE: What did you DISLIKE about the monitor settings? E.g. potential problems, limitations?

[Distracted Co2 cloud could not synchronize with Co2]

**Participant #8.1**

POSITIVE: What did you LIKE about the monitor settings? E.g., particular strengths?

[Realisitc setting]

NEGATIVE: What did you DISLIKE about the monitor settings? E.g. potential problems, limitations?

[Did not look at visual patient] [numerical values relatively too small]

**Participant #8.2**

POSITIVE: What did you LIKE about the monitor settings? E.g., particular strengths?

[CO2 display is very clear] [allows quick response]

NEGATIVE: What did you DISLIKE about the monitor settings? E.g. potential problems, limitations?

[Numbers are missing] [as a single monitor would be too little]

**Participant #9.1**

POSITIVE: What did you LIKE about the monitor settings? E.g., particular strengths?

[new]

NEGATIVE: What did you DISLIKE about the monitor settings? E.g. potential problems, limitations?

[training] [if more experience would like it better] [looked at numbers] [tried to use visual patient]

**Participant #9.2**

POSITIVE: What did you LIKE about the monitor settings? E.g., particular strengths?

[heart pulsation visual patient good]

NEGATIVE: What did you DISLIKE about the monitor settings? E.g. potential problems, limitations?

[curves and numbers smaller] [visual patient heart very small] [when vital parameter low not well visible] [mostly looked at numbers]

**Participant #10.1**

POSITIVE: What did you LIKE about the monitor settings? E.g., particular strengths?

[split monitoring helps with focus][ and increases attention]

NEGATIVE: What did you DISLIKE about the monitor settings? E.g. potential problems, limitations?

[Numbers and ranks are missing] [assessment of the severity of the situation is difficult for this reason]

**Participant #10.2**

POSITIVE: What did you LIKE about the monitor settings? E.g., particular strengths?

[it was interesting to compare Visual patient with the conventional setting] [see things quick] [listens to audio a lot] [ST-Segment better visible with visual patient]

NEGATIVE: What did you DISLIKE about the monitor settings? E.g. potential problems, limitations?

[in crisis very small changes in vital signs] [some of the reactions of the visual patient weren’t logic (e.g. heart rate appeared to be very high, even with 80 bpm)] [small changes missing in visual patient] [level of the problem is difficult to estimate]

**Participant #11.1**

POSITIVE: What did you LIKE about the monitor settings? E.g., particular strengths?

[integration of visual aspects and numerical values] [temperature display very good]

NEGATIVE: What did you DISLIKE about the monitor settings? E.g. potential problems, limitations?

[in the case of multiple pathological values, the visual patient irritates more than it helps]

**Participant #11.2**

POSITIVE: What did you LIKE about the monitor settings? E.g., particular strengths?

[quick overview of the circulatory functions] [especially oxygenation and body temperature well illustrated] [orientation help]

NEGATIVE: What did you DISLIKE about the monitor settings? E.g. potential problems, limitations?

[visual difference in rapid changes confusing, especially hemodynamics] [additional display can also be additionally confusion] [alarm fatigue, overstimulation]

**Participant #12.1**

POSITIVE: What did you LIKE about the monitor settings? E.g., particular strengths?

[individual values are first noticed in the visual patient]

NEGATIVE: What did you DISLIKE about the monitor settings? E.g. potential problems, limitations?

[Nothing]

**Participant #12.2**

POSITIVE: What did you LIKE about the monitor settings? E.g., particular strengths?

[individual vital parameter values detected first in visual patient]

NEGATIVE: What did you DISLIKE about the monitor settings? E.g. potential problems, limitations?

[too much information at once in the emergency situation] [overload] [ST-Elevation too small]

**Participant #13.1**

POSITIVE: What did you LIKE about the monitor settings? E.g., particular strengths?

[I like it] [visual patient often faster visual changes visible than with numbers or curves]

NEGATIVE: What did you DISLIKE about the monitor settings? E.g. potential problems, limitations?

[Nothing]

**Participant #13.2**

POSITIVE: What did you LIKE about the monitor settings? E.g., particular strengths?

[fast] [information simplified by visual patient] [still sufficient additional information by conventional monitoring]

NEGATIVE: What did you DISLIKE about the monitor settings? E.g. potential problems, limitations?

[Info partly displayed a bit small]

**Participant #14.1**

POSITIVE: What did you LIKE about the monitor settings? E.g., particular strengths?

[was helpful to see changes faster]

NEGATIVE: What did you DISLIKE about the monitor settings? E.g. potential problems, limitations?

[numbers were small]

**Participant #14.2**

POSITIVE: What did you LIKE about the monitor settings? E.g., particular strengths?

[additional info by visual patient] [supplementary info helpful]

NEGATIVE: What did you DISLIKE about the monitor settings? E.g. potential problems, limitations?

[no ranges at visual patient, only 0 or 1]

**Participant #15.1**

POSITIVE: What did you LIKE about the monitor settings? E.g., particular strengths?

[it gives you a picture for different things]

NEGATIVE: What did you DISLIKE about the monitor settings? E.g. potential problems, limitations?

[the system is new so we were just not used to it and that’s why it was difficult sometimes] [with some time could get used to it]

**Participant #15.2**

POSITIVE: What did you LIKE about the monitor settings? E.g., particular strengths?

[as a supplement to normal monitoring it provides a good overview] [split screen ist a great addition to the existing numerical values]

NEGATIVE: What did you DISLIKE about the monitor settings? E.g. potential problems, limitations?

-> Nothing entered

**Participant #16.1**

POSITIVE: What did you LIKE about the monitor settings? E.g., particular strengths?

[I prefer the split monitor, visual patient as first initial diagnosis – quanitfication via conventional monitoring]

NEGATIVE: What did you DISLIKE about the monitor settings? E.g. potential problems, limitations?

[visual patient alone too undifferentiated] [complicates therapy in some cases] [less space on monitor]

**Participant #16.2**

POSITIVE: What did you LIKE about the monitor settings? E.g., particular strengths?

[split monitor is good]

NEGATIVE: What did you DISLIKE about the monitor settings? E.g. potential problems, limitations?

[avatar needs practice] [too little information in the avatar (blood pressure values etc)] [SpO2 is undifferentiated by color (degree of hypoxia is unilateral)] [tube in mouth is missing in intubated patients] [relaxation is not completely clear] [visual patient alone without sound is unsatisfactory]

**Participant #17.1**

POSITIVE: What did you LIKE about the monitor settings? E.g., particular strengths?

[visually very representative] [visual patient very realistic] [good representations with vital signs]

NEGATIVE: What did you DISLIKE about the monitor settings? E.g. potential problems, limitations?

[limits unclear, therefore I did not know when the visual patient turns blue or where CO2 levels are]

**Participant #17.2**

POSITIVE: What did you LIKE about the monitor settings? E.g., particular strengths?

[ABCDE quickly clear in split setting due to the figure] [effective] [fine adjustments possible using the numbers in split screen]

NEGATIVE: What did you DISLIKE about the monitor settings? E.g. potential problems, limitations?

[perhaps too little focus on the patient]

**Participant #18.1**

POSITIVE: What did you LIKE about the monitor settings? E.g., particular strengths?

[color supports][ to perceive changes quickly] [colors seem more drastic than the numbers alone, so you feel you act faster or want to act faster]

NEGATIVE: What did you DISLIKE about the monitor settings? E.g. potential problems, limitations?

[Nothing]

**Participant #18.2**

POSITIVE: What did you LIKE about the monitor settings? E.g., particular strengths?

[I like the combination of new and old monitoring, so we could start without delay or having to think about anything] [visual patient helped putting the picture together]

NEGATIVE: What did you DISLIKE about the monitor settings? E.g. potential problems, limitations?

[visual patient is too large on the screen] [I’d rather see more numbers] [I barely looked at the visual patient]

**Participant #19.1**

POSITIVE: What did you LIKE about the monitor settings? E.g., particular strengths?

[visual patient provides an overview]

NEGATIVE: What did you DISLIKE about the monitor settings? E.g. potential problems, limitations?

[does not provide any additional information] [information from dummy inaccurate] [unclear when action needed] [takes up too much space]

**Participant #19.2**

POSITIVE: What did you LIKE about the monitor settings? E.g., particular strengths?

[all parameters visible and you can still visualize the patient] [Saturation quickly visible with visual patient]

NEGATIVE: What did you DISLIKE about the monitor settings? E.g. potential problems, limitations?

[arrangements were not as usual]

**Participant #20.1**

POSITIVE: What did you LIKE about the monitor settings? E.g., particular strengths?

[Cardiac problems (eg myocardial infarction) better recognized]

NEGATIVE: What did you DISLIKE about the monitor settings? E.g. potential problems, limitations?

[well trained with conventional monitoring, therefore it could be possible that the visual patient would not be noticed] [values and curves very small in split screen]

**Participant #20.2**

POSITIVE: What did you LIKE about the monitor settings? E.g., particular strengths?

[since the numbers had different colors, everything was very visble]

NEGATIVE: What did you DISLIKE about the monitor settings? E.g. potential problems, limitations?

[too much information in the picture]

**Participant #21.1**

POSITIVE: What did you LIKE about the monitor settings? E.g., particular strengths?

[good short overview of vital parameters] [alarms in case of serious problems]

NEGATIVE: What did you DISLIKE about the monitor settings? E.g. potential problems, limitations?

[visualization lacks numerical value] [display reduces attention from conventional monitor]

**Participant #21.2**

POSITIVE: What did you LIKE about the monitor settings? E.g., particular strengths?

[the colors help to see more quickly which body funtions are affected]

NEGATIVE: What did you DISLIKE about the monitor settings? E.g. potential problems, limitations?

[too much information at a glance, especially in emergency situations] [visual patient is shown small in this setting]

**Participant #22.1**

POSITIVE: What did you LIKE about the monitor settings? E.g., particular strengths?

[color good] [can be used well for a short time for quick orientation] [in combination with conventional monitoring well imaginable]

NEGATIVE: What did you DISLIKE about the monitor settings? E.g. potential problems, limitations?

[visual patient alone too little monitoring for me] [association to the patient situation is too little] [tending to endanger the patient]

**Participant #22.2**

POSITIVE: What did you LIKE about the monitor settings? E.g., particular strengths?

[I like the split screen best, because I have the monitor that I feel very familiar with and that gives me the detail of information I need] [interesting visual addition to put emphasis on certain aspects]

NEGATIVE: What did you DISLIKE about the monitor settings? E.g. potential problems, limitations?

[bigger monitor would improve the clarity]

**Participant #23.1**

POSITIVE: What did you LIKE about the monitor settings? E.g., particular strengths?

[I liked this option, but I focus more on conventional monitoring]

NEGATIVE: What did you DISLIKE about the monitor settings? E.g. potential problems, limitations?

[not used to visual patient] [visual patient made me feel insecure] [it is not immediately visible and clear to me] [visual patient is an unknown system]

**Participant #23.2**

POSITIVE: What did you LIKE about the monitor settings? E.g., particular strengths?

[in split screen one sees the values in a known way]

NEGATIVE: What did you DISLIKE about the monitor settings? E.g. potential problems, limitations?

[in split screen the visual patient can be distracting as only certain parameters are intrepreted] [no tendency shown and thus no early intervention] [treatment with the visual patient only difficult, because of the unknown system]

**Participant #24.1**

POSITIVE: What did you LIKE about the monitor settings? E.g., particular strengths?

[good supplement to quickly record vital signs] [very simple system] [I love it]

NEGATIVE: What did you DISLIKE about the monitor settings? E.g. potential problems, limitations?

[space on my screen is getting smaller and smaller] [needs appropriate monitor size]

**Participant #24.2**

POSITIVE: What did you LIKE about the monitor settings? E.g., particular strengths?

[the two system complement each other well] [Most of the features (e.g. heart rate) are intuitive] [easy] [ fast to capture]

NEGATIVE: What did you DISLIKE about the monitor settings? E.g. potential problems, limitations?

[The blood pressure feature was not easy to understand for me.]

**Participant #25.1**

POSITIVE: What did you LIKE about the monitor settings? E.g., particular strengths?

[The temperature change was well represented]

NEGATIVE: What did you DISLIKE about the monitor settings? E.g. potential problems, limitations?

[Visual patient: Head too large in contrast to heart/lung]

**Participant #25.2**

POSITIVE: What did you LIKE about the monitor settings? E.g., particular strengths?

[Overview of relevant parameters at a glance through Visual Patient]

NEGATIVE: What did you DISLIKE about the monitor settings? E.g. potential problems, limitations?

[Changes in the visual patient only as a very rough guideline] [difficult to interpret if only little experience with visual patient] [proportions not clearly arranged]

**Participant #26.1**

POSITIVE: What did you LIKE about the monitor settings? E.g., particular strengths?

[I really liked the combination] [the Bis monitoring and temperature as well as the relaxation is easier to see with the visual version] [The visual version is very useful for a first impression of the patient]

NEGATIVE: What did you DISLIKE about the monitor settings? E.g. potential problems, limitations?

[if i want to see the details or parameter more precisely i prefer the ""usual"" monitor view] [I prefer the ekg in the ""usual"" way because you can see the changes quicker and itˋs easier to notice it.]

**Participant #26.2**

POSITIVE: What did you LIKE about the monitor settings? E.g., particular strengths?

[The Visual Patient quickly makes it clear that something is wrong] [Probably one gets used to it relatively well]

NEGATIVE: What did you DISLIKE about the monitor settings? E.g. potential problems, limitations?

[Initially confusing, too much information on one monitor] [Absoluta of the Visual Patient (too high, too low) not relevant for me, as long as not internalized, which values are behind it] [Trends are often more important than current values] [Quantification of the problem with numbers additionally important]

**Participant #27.1**

POSITIVE: What did you LIKE about the monitor settings? E.g., particular strengths?

[It is able to highlight details in the patient's overall problem such as: increased temperature with orange stripes/cardiac ischemia with black dot]

NEGATIVE: What did you DISLIKE about the monitor settings? E.g. potential problems, limitations?

[Missing numerical values that show a progression or improvement/deterioration of a parameter] [One must be familiar with the graph to interpret it or is susceptible to subjectivity]

**Participant #27.2**

POSITIVE: What did you LIKE about the monitor settings? E.g., particular strengths?

[Super cute] [Temperature monitoring is helpful]

NEGATIVE: What did you DISLIKE about the monitor settings? E.g. potential problems, limitations?

[Not too useful without values]

**Participant #28.1**

POSITIVE: What did you LIKE about the monitor settings? E.g., particular strengths?

[Everything was visible at a glance.]

NEGATIVE: What did you DISLIKE about the monitor settings? E.g. potential problems, limitations?

[Visual patient: Too much information that is partially irritating.]

**Participant #28.2**

POSITIVE: What did you LIKE about the monitor settings? E.g., particular strengths?

[Combination of visual patient and conventional qualitative and quantitative (numeric) values are combined]

NEGATIVE: What did you DISLIKE about the monitor settings? E.g. potential problems, limitations?

[Additional visualization might occupy space/visibility of numeric values.]

**Participant #29.1**

POSITIVE: What did you LIKE about the monitor settings? E.g., particular strengths?

[I am used to the numbers of conventional monitoring and find it very helpful for me to see both.]

NEGATIVE: What did you DISLIKE about the monitor settings? E.g. potential problems, limitations?

[I was so fixated on the monitor that I hardly looked at the patient]

**Participant #29.2**

POSITIVE: What did you LIKE about the monitor settings? E.g., particular strengths?

[Clear numerical values help with the conventional monitor] [Visual Patient summarizes everything at a glance] [Overall, probably faster overview with both and][ faster noticing of changes]

NEGATIVE: What did you DISLIKE about the monitor settings? E.g. potential problems, limitations?

[Visual patient takes some getting used to,][not entirely intuitive at first.]

**Participant #30.1**

POSITIVE: What did you LIKE about the monitor settings? E.g., particular strengths?

[First look at the visual patient and you have an idea what to look for] [Second look goes to the numbers to then take the necessary measures]

NEGATIVE: What did you DISLIKE about the monitor settings? E.g. potential problems, limitations?

[In the beginning you have to get used and adapt to the new circumstances]

**Participant #30.2**

POSITIVE: What did you LIKE about the monitor settings? E.g., particular strengths?

[Combination of graphics and values] [Improves overall image]

NEGATIVE: What did you DISLIKE about the monitor settings? E.g. potential problems, limitations?

[Screen very full] [Curves very small and short] [Visual patient very large compared to the curves]

**Participant #31.1**

POSITIVE: What did you LIKE about the monitor settings? E.g., particular strengths?

[Intuitively understandable] [More impressive through color change than pure numbers]

NEGATIVE: What did you DISLIKE about the monitor settings? E.g. potential problems, limitations?

[Only with visual patient No tendencies recognizable (only too high/low)] [With split screen I ignore visual patient, since used to conventional monitor]

**Participant #31.2**

POSITIVE: What did you LIKE about the monitor settings? E.g., particular strengths?

[numbers and visual patient is intense / statements more powerful]

NEGATIVE: What did you DISLIKE about the monitor settings? E.g. potential problems, limitations?

[Nothing]

**Participant #32.1**

POSITIVE: What did you LIKE about the monitor settings? E.g., particular strengths?

[Visual patient is quicker to perceive significant changes] [But in combination with the normal vital parameters it is very good]

NEGATIVE: What did you DISLIKE about the monitor settings? E.g. potential problems, limitations?

[You still have to get used to it.]

**Participant #32.2**

POSITIVE: What did you LIKE about the monitor settings? E.g., particular strengths?

Combination of the respective advantages. Visual patient: fast acquisition with only one value deviation. Conventional: clearer presentation of the combination]

NEGATIVE: What did you DISLIKE about the monitor settings? E.g. potential problems, limitations?

[Visual patient attracts a lot of attention] [visual patient presents the information in a confusing way, especially when several abnormal values are combined]

**Participant #33.1**

POSITIVE: What did you LIKE about the monitor settings? E.g., particular strengths?

[Intuitive additional information of the temperature by the male, which is otherwise often overlooked as a single number] [You can see the stress of the patient very well on the male]

NEGATIVE: What did you DISLIKE about the monitor settings? E.g. potential problems, limitations?

[for the interpretation helps me personally the classic representation better] [If the visual patient occupies a lot of screen space, the classic monitoring may be somewhat displaced or too small to remain clear] [No gradations are visible on the visual patient (e.g. height of the BP, depth of anesthesia), so it is not sufficient as the only monitor]

**Participant #33.2**

POSITIVE: What did you LIKE about the monitor settings? E.g., particular strengths?

[Combination of standard curves and colored visual patient help to detect][ and respond to vital sign changes faster]

NEGATIVE: What did you DISLIKE about the monitor settings? E.g. potential problems, limitations?

[low CO2 is noticed late with the small bubble]

**Participant #34.1**

POSITIVE: What did you LIKE about the monitor settings? E.g., particular strengths?

[The visual patient gives a good overview] [I liked the combination]

NEGATIVE: What did you DISLIKE about the monitor settings? E.g. potential problems, limitations?

Nothing reported

**Participant #34.2**

POSITIVE: What did you LIKE about the monitor settings? E.g., particular strengths?

[Good global overview of vital Status]

NEGATIVE: What did you DISLIKE about the monitor settings? E.g. potential problems, limitations?

[Dynamic of Change in vital Status is delayed in recognition due to abstract Show and Switch of e.g. Color and arterial pressure]

**Participant #35.1**

POSITIVE: What did you LIKE about the monitor settings? E.g., particular strengths?

[One recognizes with the visual patient at a glance a cyanosis respectively saturation drop (stings the eye)] [has beside the relative change at the same time the absolute changes of the numbers]

NEGATIVE: What did you DISLIKE about the monitor settings? E.g. potential problems, limitations?

[Can lose the overview beside the numbers however somewhat, since it is something additional to look at]

**Participant #35.2**

POSITIVE: What did you LIKE about the monitor settings? E.g., particular strengths?

[Both available, can decide for yourself where to look, you can quickly see from the visual patient that something is not good, quick impression]

NEGATIVE: What did you DISLIKE about the monitor settings? E.g. potential problems, limitations?

[But would need practice for interpretation.] [Still very unfamiliar,][ no concrete values are displayed]

**Participant #36.1**

POSITIVE: What did you LIKE about the monitor settings? E.g., particular strengths?

[I can choose what I want to take numbers or the visual patient]

NEGATIVE: What did you DISLIKE about the monitor settings? E.g. potential problems, limitations?

[In the visual patient there is too little information][ and too few tendencies for me to foresee paths.]

**Participant #36.2**

POSITIVE: What did you LIKE about the monitor settings? E.g., particular strengths?

[Some values are recorded more quickly with visual patient, especially Temperature, CO2!] [I like it]

NEGATIVE: What did you DISLIKE about the monitor settings? E.g. potential problems, limitations?

[With only visual patient the tendencies are missing]

**Participant #37.1**

POSITIVE: What did you LIKE about the monitor settings? E.g., particular strengths?

[Simple and] [quick to grasp situation]

NEGATIVE: What did you DISLIKE about the monitor settings? E.g. potential problems, limitations?

[Learning phase is urgently needed]

**Participant #37.2**

POSITIVE: What did you LIKE about the monitor settings? E.g., particular strengths?

[Integrated information from both assessments]

NEGATIVE: What did you DISLIKE about the monitor settings? E.g. potential problems, limitations?

[Delay due to too much visual information collection]

**Participant #38.1**

POSITIVE: What did you LIKE about the monitor settings? E.g., particular strengths?

[I found the visualization next to the ECG good, because you saw in the visual patient that there is ischemia] [If you know where to look, that can be a good addition]

NEGATIVE: What did you DISLIKE about the monitor settings? E.g. potential problems, limitations?

[The visual patient is upside down for me. Head up would be more intuitive for me] [Heart and lung parameters are represented very small and you have to look closely and especially know that something can change there] [It is stressful when the patient is blinking and you don't know exactly what it is, there seem to be several problems at the same time] [At the moment still difficult but with potential]

**Participant #38.2**

POSITIVE: What did you LIKE about the monitor settings? E.g., particular strengths?

[It is exciting, you can include it] [It is nice to look at] [The different colors are good]

NEGATIVE: What did you DISLIKE about the monitor settings? E.g. potential problems, limitations?

[In emergency situations is disturbing for me] [HF and RR are difficult to distinguish] [It is then also missing the numbers] [however, it is irritating for me] [I can't differentiate it yet which is which.][ It distracts me then] [The clinic is prioritized, but if the visual patient does not correlate, such as with the curves.... it is confusing for me]

**Participant #39.1**

POSITIVE: What did you LIKE about the monitor settings? E.g., particular strengths?

[Color coding of the Visual Patient][ allows faster detection of the situation]

NEGATIVE: What did you DISLIKE about the monitor settings? E.g. potential problems, limitations?

[Familiar curves may have too little space in the split screen, in particular the arterial curve would have to be wider, as otherwise only numbers would suffice (e.g. 8/4 mode)]

**Participant #39.2**

POSITIVE: What did you LIKE about the monitor settings? E.g., particular strengths?

[That I can see both. Can so better see a connection]

NEGATIVE: What did you DISLIKE about the monitor settings? E.g. potential problems, limitations?

[I was missing the numbers.] [I do not look at the numbers but only at the visual patient]

**Participant #40.1**

POSITIVE: What did you LIKE about the monitor settings? E.g., particular strengths?

[Split screen: fast qualitative capture, then more accurate quantitative capture with conventional monitor]

NEGATIVE: What did you DISLIKE about the monitor settings? E.g. potential problems, limitations?

[Split screen: Reduction of the conventional monitor, omission of the saturation curve?]

**Participant #40.2**

POSITIVE: What did you LIKE about the monitor settings? E.g., particular strengths?

[Combi: Quick recognition e.g. by senior physician who visits the situation or observes from the background] [Normal monitoring: focus more on curve and value]

NEGATIVE: What did you DISLIKE about the monitor settings? E.g. potential problems, limitations?

[Combi:Ev distraction,][ numerical values smaller] [Norm monitoring: Overlooking possible because more focus on acoustics]

**Participant #41.1**

POSITIVE: What did you LIKE about the monitor settings? E.g., particular strengths?

[Usual values displayed, graphical extension] [if necessary More safety]

NEGATIVE: What did you DISLIKE about the monitor settings? E.g. potential problems, limitations?

[Nothing]

**Participant #41.2**

POSITIVE: What did you LIKE about the monitor settings? E.g., particular strengths?

[Have numbers with on the monitor but visualizations from visual patient shows states partially faster] [Find visual patient+conventional monitor as a win]

NEGATIVE: What did you DISLIKE about the monitor settings? E.g. potential problems, limitations?

Nothing recorded

**Participant #42.1**

POSITIVE: What did you LIKE about the monitor settings? E.g., particular strengths?

[Additional control of the patient] [Makes the patient visually noticeable.]

NEGATIVE: What did you DISLIKE about the monitor settings? E.g. potential problems, limitations?

[Can be distracting, especially in hectic situations] [Possibly also just because not used to it.]

**Participant #42.2**

POSITIVE: What did you LIKE about the monitor settings? E.g., particular strengths?

[Both available, short overview vs view all data fields]

NEGATIVE: What did you DISLIKE about the monitor settings? E.g. potential problems, limitations?

[Ekg smaller] [arterial curve smaller] [no raw eeg (no curve)]

**Participant #43.1**

POSITIVE: What did you LIKE about the monitor settings? E.g., particular strengths?

[CO2 strongly in focus (better visible)]

NEGATIVE: What did you DISLIKE about the monitor settings? E.g. potential problems, limitations?

[arrhythmia detected late] [blood pressure in visual patient irritating without value]

**Participant #43.2**

POSITIVE: What did you LIKE about the monitor settings? E.g., particular strengths?

[Good for blood pressure perception and arrhythmia] [The combination could be useful]

NEGATIVE: What did you DISLIKE about the monitor settings? E.g. potential problems, limitations?

[the curves are missing, such as The Spo2 curve] [Thorax depicted too small] [V.cava representation initially unclear]

**Participant #44.1**

POSITIVE: What did you LIKE about the monitor settings? E.g., particular strengths?

[If someone knows Visual Patient, it can be that you quickly get a good eye (good overview) of how the patient is doing]

NEGATIVE: What did you DISLIKE about the monitor settings? E.g. potential problems, limitations?

[Personally, I did not pay attention to the Visual Patient because I am very fixated -as usual- on the conventional monitoring]

**Participant #44.2**

POSITIVE: What did you LIKE about the monitor settings? E.g., particular strengths?

[I liked that you still have a visual representation of the current patient situation]

NEGATIVE: What did you DISLIKE about the monitor settings? E.g. potential problems, limitations?

[Due to the fact that I see the system for the first time, I find it difficult to interpret all the information correctly]

**Participant #45.1**

POSITIVE: What did you LIKE about the monitor settings? E.g., particular strengths?

[Combination I find good though]

NEGATIVE: What did you DISLIKE about the monitor settings? E.g. potential problems, limitations?

[Avatar should look more like a human] [Too small numeric values]

**Participant #45.2**

POSITIVE: What did you LIKE about the monitor settings? E.g., particular strengths?

[Good addition to get a quick overview]

NEGATIVE: What did you DISLIKE about the monitor settings? E.g. potential problems, limitations?

[Too many visual impressions]

**Participant #46.1**

POSITIVE: What did you LIKE about the monitor settings? E.g., particular strengths?

[Visual information can be integrated]

NEGATIVE: What did you DISLIKE about the monitor settings? E.g. potential problems, limitations?

[Misinterpretation]

**Participant #46.2**

POSITIVE: What did you LIKE about the monitor settings? E.g., particular strengths?

[Faster overall view of patient situation] [Easier interpretation of patient values through visual patient]

NEGATIVE: What did you DISLIKE about the monitor settings? E.g. potential problems, limitations?

[Difficult to recognize deterioration from visualization, as there is a threshold value (no quantification)] [visualization distracts from numerical values]

**Participant #47.1**

POSITIVE: What did you LIKE about the monitor settings? E.g., particular strengths?

[For diagnostics, I was able to get information from both monitors] [It was helpful.]

NEGATIVE: What did you DISLIKE about the monitor settings? E.g. potential problems, limitations?

[Putting two sources of information together into one picture was a bit exhausting.]

**Participant #47.2**

POSITIVE: What did you LIKE about the monitor settings? E.g., particular strengths?

[Integration of all values on one avatar]

NEGATIVE: What did you DISLIKE about the monitor settings? E.g. potential problems, limitations?

[Many inputs! ][Getting used to is always exhausting!]

**Participant #48.1**

POSITIVE: What did you LIKE about the monitor settings? E.g., particular strengths?

[Nothing]

NEGATIVE: What did you DISLIKE about the monitor settings? E.g. potential problems, limitations?

[Very confusing] [lacking information quantification with the visual patient] [not sure about the added benefit if conventional monitoring is available]

**Participant #48.2**

POSITIVE: What did you LIKE about the monitor settings? E.g., particular strengths?

[Nothing]

NEGATIVE: What did you DISLIKE about the monitor settings? E.g. potential problems, limitations?

[Of course I find it unfamiliar] [the visualization too inaccurate]

**Participant #49.1**

POSITIVE: What did you LIKE about the monitor settings? E.g., particular strengths?

[Quick assessment~~][~~ of the situation at a glance with additional exact numerical values]

[Especially blood pressure is quickly visible in the figure]

NEGATIVE: What did you DISLIKE about the monitor settings? E.g. potential problems, limitations?

[Nothing]

**Participant #49.2**

POSITIVE: What did you LIKE about the monitor settings? E.g., particular strengths?

[Splitscreen: Combination of the visual like colors and the known numbers and curves.]

NEGATIVE: What did you DISLIKE about the monitor settings? E.g. potential problems, limitations?

[The Visual patient without values and curves]

**Participant #50.1**

POSITIVE: What did you LIKE about the monitor settings? E.g., particular strengths?

[Good combination of the simple visualizations (visual patient) to the ""complex"" Standard monitoring

NEGATIVE: What did you DISLIKE about the monitor settings? E.g. potential problems, limitations?

[Possibly distracted by the visual patient - subjectively he gets more attention in my opinion]

**Participant #50.2**

POSITIVE: What did you LIKE about the monitor settings? E.g., particular strengths?

[Information in the Visual Patient at a glance~~] [~~quantification of values via conventional monitor]

NEGATIVE: What did you DISLIKE about the monitor settings? E.g. potential problems, limitations?

[A lot of information at once] [No finer graduation in the Visual Patient]

**Participant #51.1**

POSITIVE: What did you LIKE about the monitor settings? E.g., particular strengths?

[I liked that the tones for heart rate and saturation were present. As well as in reality]

NEGATIVE: What did you DISLIKE about the monitor settings? E.g. potential problems, limitations?

[It was confusing for me in parts. On the one hand to pay attention to the visual patient and then at the same time to the parameters.]

**Participant #51.2**

POSITIVE: What did you LIKE about the monitor settings? E.g., particular strengths?

[It could also be good for practice]

NEGATIVE: What did you DISLIKE about the monitor settings? E.g. potential problems, limitations?

[I do not find it bad, but we are just used to the number] [unfortunately, one is not accustomed to the separate screen]

**Participant #52.1**

POSITIVE: What did you LIKE about the monitor settings? E.g., particular strengths?

[Relief: no fixation on the numbers is more relaxed

NEGATIVE: What did you DISLIKE about the monitor settings? E.g. potential problems, limitations?

[Risk of overlooking something important]

**Participant #52.2**

POSITIVE: What did you LIKE about the monitor settings? E.g., particular strengths?

[More information,][ presented in a simple/abstract way]

NEGATIVE: What did you DISLIKE about the monitor settings? E.g. potential problems, limitations?

[Split screen leads to smaller display of detailed values,][ one rather looks at the usual numerical values]
